# Burns, hypertrophic scar and galactorrhea

**DOI:** 10.5249/jivr.v5i2.314

**Published:** 2013-06

**Authors:** Hamid Karimi, Samad Nourizad, Mahnoush Momeni, Hosein Rahbar, Mazdak Momeni, Khosro Farhadi

**Affiliations:** ^*a*^ Faculty of Medicine, Tehran University of Medical Sciences,Tehran, Iran.; ^*b*^Baylor College of Medicine, Houston, Texas, USA.; ^*c*^Department of Anesthesiology, Imam Reza Hospital, Kermanshah University of Medical Sciences, Kermanshah, Iran.

**Keywords:** Burns, Hypertrophic scar, Galactorrhea

## Abstract

An 18-year old woman was admitted to Motahari Burn Center suffering from 30% burns. Treatment modalities were carried out for the patient and she was discharged after 20 days. Three to four months later she developed hypertrophic scar on her chest and upper limbs. At the same time she developed galactorrhea in both breasts and had a disturbed menstrual cycle four months post-burn. On investigation, we found hyperprolactinemia and no other reasons for the high level of prolactin were detected.

She received treatment for both the hypertrophic scar and the severe itching she was experiencing.

After seven months, her prolactin level had decreased but had not returned to the normal level. It seems that refractory hypertrophic scar is related to the high level of prolactin in burns patients.

## Introduction

This is a case report of galactorrhea and refractory hypertrophic burn scar that developed in one of our patients. Galactorrhea is a very rare occurrence in burns patients and in the past ten years we had not any other patients with this problem.

## Case Study

**Figure 1:**
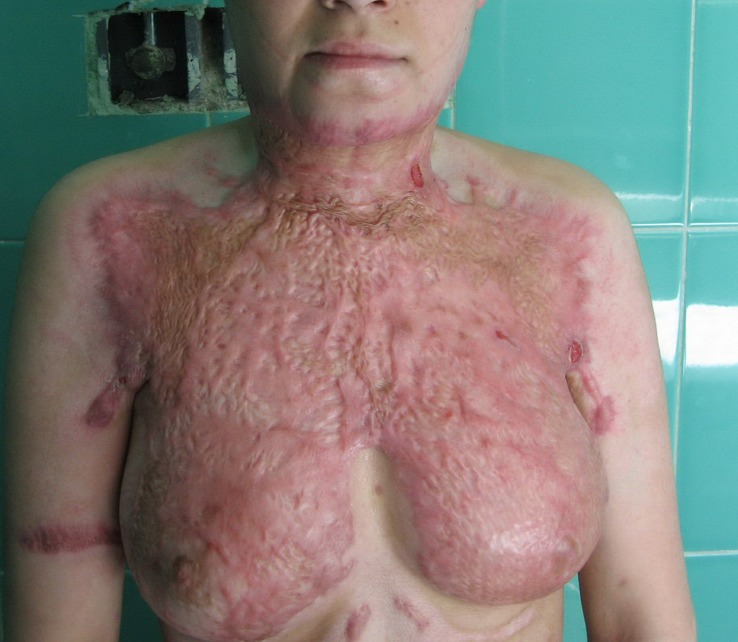
The patient with severe hypertrophic burn scar

An 18-year-old housewife with no previous significant medical history presented to the emergency burns department. On physical examination, she had 30% superficial and deep partial thickness and full thickness burns (second and third degree) on the anterior and posterior surface of her trunk, right and left upper limbs, her face, neck and ears (Figure 1). The burns had been caused by an accident with kerosene. Seven days after admission some parts of her wound showed signs of sepsis, i.e. marginal edema, cellulitis and burn wound focal gangrene.

**Figure 2:**
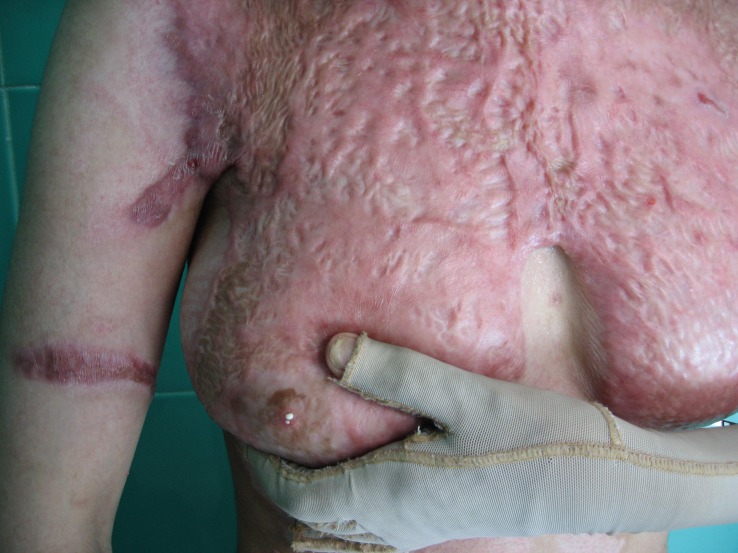
Note the milk on her nipple

For treatment, burn wound biopsy, tissue culture and antibiotic therapy were begun. Some parts of her partial thickness burn were treated with dressing and spontaneous epithelialization and other parts were grafted. Three to four months after discharge she was readmitted with galactorrhea (Figure 2).

Although her premorbid menstrual cycle was normal, she developed a disturbed menstrual cycle four months post-burn.

Serum prolactin levels: Radioimmunoassay study of various hormonal levels was performed. Thyroid function test showed no abnormality and FSH and LH levels were normal.

**Figure 3:**
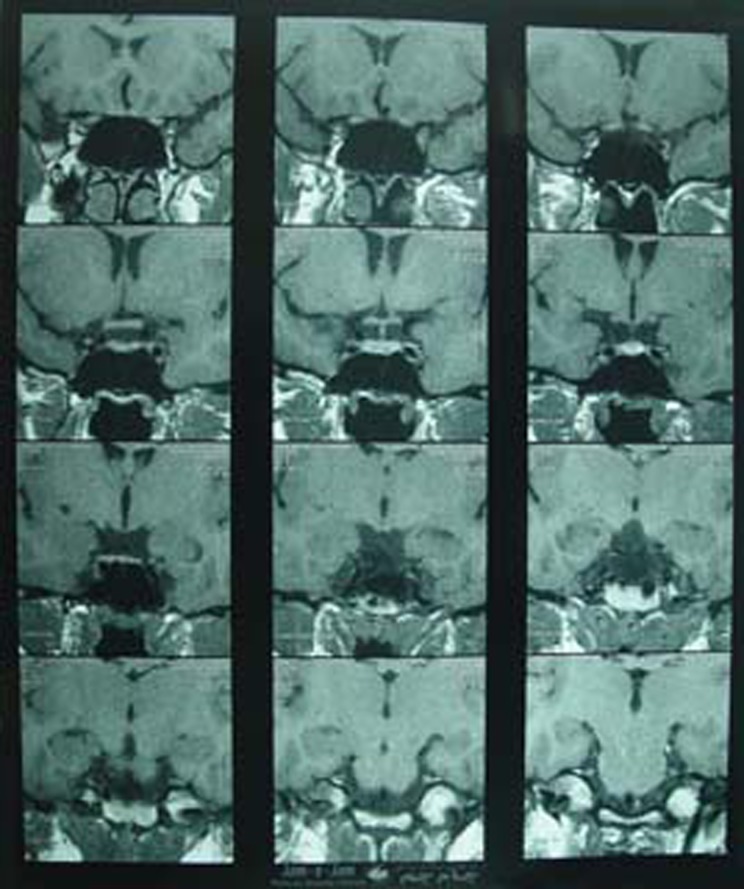
The MRI of patient’s brain and pituitary gland that shows no pituitary tumor

**Figure 4:**
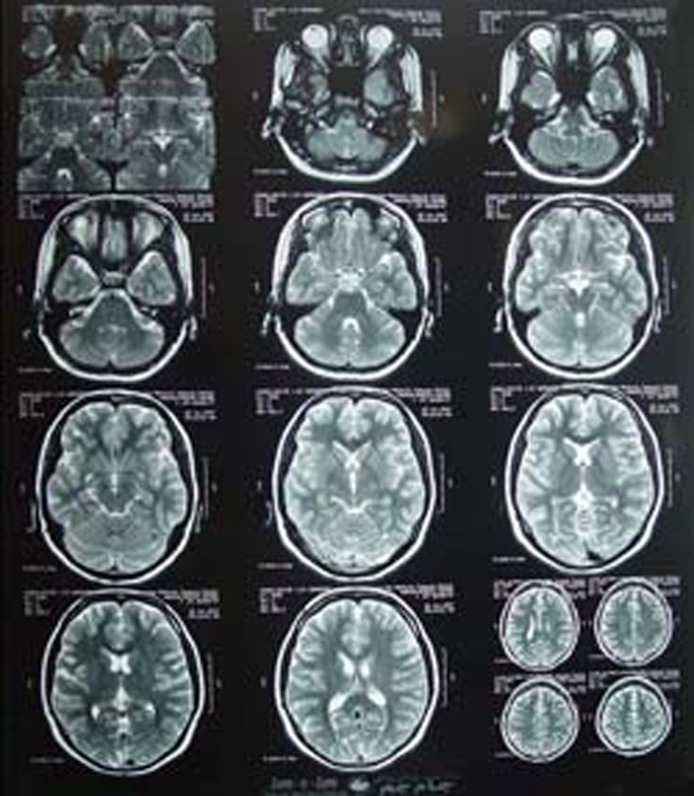
The MRI of patient’s brain and pituitary gland that shows no pituitary tumor

Other cause of galactorrhea, such as pituitary adenoma (Figure 3,4), hypothyroidism, epileptic seizure, renal disease, antiarrhythmic drugs^6^ and non-pituitary prolactin-producing tumors, were excluded by the appropriate investigations. This patient was not on contraceptives, antidepressants or antihypertensive medication. She was not obese (BMI=19.05). Hypertrophic scars and keloids are the most common sequel of deep burns. The patient developed hypertrophic scars despite postoperative preventive treatment, such as pressure garments, silicon sheet and steroid injections (Figure 1).

She had a very intense episode of itching. Seven months after the burn accident, the prolactin level was lower, but had not returned to the normal level. Her galactorrhea began 3 months after wound closure. Bromocriptin was prescribed for the patient and galactorrhea gradually subsided during the seven-month post-burn period. At this time, the hypertrophic scar responded to treatment and subsided too.

## Discussion

Galactorrhea or galactorrhoea is the spontaneous flow of milk from the breast, unassociated with childbirth or nursing. Galactorrhea is reported to occur in 5%-32% percent of women, much of the difference in reported incidence can be attributed to different definitions of galactorrhea.^3^ Galactorrhea also occurs in males, newborn infants and adolescents of both sexes. ^4^ Although frequently benign, it may be caused by serious underlying conditions and should be properly investigated.^5^ It can be due to dysregulation of certain hormones or local causes such as excessive nipple stimulation. Hormonal causes most frequently associated with galactorrhea are hyperprolactinemia and thyroid conditions with elevated levels of TSH or TRH hormones. Nevertheless no obvious cause is found in about 50% of cases.^3^

Lactation requires the presence of estrogen, progesterone and prolactin, and the evaluation of galactorrhea includes an elicitation of the patient’s history for various medications or foods (methyldopa, opiates, anti-psychotics, serotonin reuptake inhibitors, as well as licorice and for behavioral causes (stress, and breast and chest wall stimulation), as well as evaluation for pregnancy, pituitary adenomas (with over production of prolactin or compression of the pituitary stalk), and hypothyroidism. Treatment is based on discontinuing of medication and reduction of stress factors and treatment of hormone irregularities and adenomas. Adenomas of the anterior pituitary are most often prolactinomas. Overproduction of prolactin leads to cessation of menstrual periods and infertility, which may be a diagnostic clue. Galactorrhea may also be caused by hormonal imbalances owing to contraceptive pills.

Galactorrhea is also a side effect associated with the use of the second-generation H2 receptor antagonist Cimetidine. Galactorrhea can be also caused by anti-psychotics that cause hyperprolactinemia by blocking dopamine receptors responsible for control of prolactin release. Of these, risperidone is the most notorious for causing this complication. Case reports suggest proton-pump inhibitors have been shown to cause galactorrhea. Galactorrhea is the pathologic non-puerperal secretion of milk. The hypothalamus has an essential role in controlling the secretion of anterior pituitary hormones, and any damage to the hypothalamus or blockage of the hypothalamic–hypophyseal portal system, such as trauma increases prolactin secretion from anterior pituitary.^2^ Another etiology of hyperprolactinemia is physiological hypersecretion such as chest wall stimulation and stress. In a burns patient, there are several factors which affect immune status. Dopamine profoundly influences the immune status and adrenal steroid secretion in burns patients. Several studies indicate that dopamine treatment may undermine an already depressed immune system. This effect appears to act via the suppression of prolactin release from the anterior pituitary gland.

Dopamine suppresses serum prolactin and DHEAS levels, thus, it is possible that the dopamine-induced suppression of prolactin is responsible for lowering DHEAS levels and the consequent suppression of the T-cell proliferative response. ^1^ This may partly be responsible for the anergic state of the immune system during severe stress. In a burns patient during the acute phase and before coverage of burn wounds, the patient is in a catabolic and hyper-catabolic state. After coverage of the wound, the patient will be in the anabolism phase and the activity of the immune system will be increased. A large number of burn scars have hypertrophic responses: these hypertrophic scars could be controlled with therapeutic strategies such as pressure garments and silicone sheets. In the Saraiya report, the burns patient who developed galactorrhea also suffered from persistent and continual development of hypertrophic scars, annoying itching and keloid formation in spite of the preventive strategies. ^2^ Their patients were obese and they believed that obese patients are more prone for developing galactorrhea and refractory hypertrophic scars. ^2^ As was mentioned above, our patient was not obese. She had galactorrhea and she suffered from persistent and continual development of hypertrophic scars even though the preventive measures were pursued vigorously. In another report, there was significant correlation between amenorrhea-galactorrhea and chest abdomen and upper limb burns. ^3^ All scars tend to worsen three to four months after burn as collagen deposition and scar contraction take place in the setting of very active anabolism and exactly at this period of time, there was a very high level of prolactin in our patient. After the first three to four months, anabolism slows down and hypertrophic scars slowly begin to reduce in size, soften and became less erythematic but the speed of this process in our patient was slow and the prolactin level in this phase remained high. As a result, we believe that a rise in prolactin levels at the time of increased immune response in burns patients may play a role in the development of refractory hypertrophic scars.
